# Examining the Role of Self-Reliance, Social Domination, Perceived Surveillance, and Customer Support with Respect to the Adoption of Mobile Banking

**DOI:** 10.3390/ijerph20053854

**Published:** 2023-02-21

**Authors:** Mohammad Asif, Mohammed Arshad Khan, Hamad Alhumoudi, Mohammad Wasiq

**Affiliations:** 1 Department of Finance, College of Administration and Financial Science, Saudi Electronic University, Riyadh 11673, Saudi Arabia; 2 Department of Accountancy, College of Administration and Financial Science, Saudi Electronic University, Riyadh 11673, Saudi Arabia; 3 College of Administration and Financial Science, Saudi Electronic University, Riyadh 11673, Saudi Arabia

**Keywords:** mobile banking, perceived surveillance, self-reliance and social domination

## Abstract

Purpose: This article aims to investigate the main drivers of mobile banking among Delhi–NCR consumers. The TAM (technological acceptance model) was used as a framework for this study. Only a few studies have looked at how online banking users in India plan to use other similar services, such as m-banking. In order to do this, a theoretical model was made using the technology acceptance model. This model was then expanded to include the factors that make m-banking users more likely to use mobile banking. These adoption factors include the feeling of being watched, the ability to do things on your own with a mobile device, social dominance, and the role of customer support as a mediator. The use of m-banking is the thing that matters. Scope: In the last two decades, digital mobile devices have become the primary preferred method of consumer communication. Throughout the past year, mobile banking has become increasingly popular. The increasing number of smartphones in use, as well as the government’s push for cashless transactions, provide an opportunity for the Indian banking industry to rapidly expand its usage of mobile and online banking. Methodology: The data were collected through a structured questionnaire distributed to 376 respondents from different sustainable investment classes. The use of convenience sampling was imposed. Structure equation modeling (SEM), reliability, convergence, discriminate validity, and model fitness were achieved through SmartPLS 3. Findings: The study found that the adoption factors had a significant impact on perceived surveillance, mobile self-reliance, and social domination and mediating role of customer support to use mobile banking. These latest findings will inform banks and financial institutions on the rise of m-banking in India, as well as provide insight into digital banking channels and add to the literature on the topic of digital banking adoption.

## 1. Introduction

Internet and the wireless revolution have altered the manner in which people communicate and engage. Technology has revolutionized the functioning and delivery of banking services by banks. Customers are less likely to visit bank branches as a result of the rapid development of ICT, cellular phones, and other mobile devices [1]. They have shifted the majority of their banking activities to be completed online. Smartphones, disruptive technology such as artificial intelligence and chatbots, and voice-based payment systems are all contributing to the shift away from traditional banking toward digital banking services. An emerging trend in mobile phone use is the expansion of its functionality beyond basic communication to include use as a means of personal expression and augmentation of one’s everyday life [2]. These days, more and more consumers rely on their mobile devices to do everything from discovering local businesses, to conducting product research, to making purchases, and managing their accounts. Mobile banking is a recent technological breakthrough [3] that has revolutionized the financial services industry by making it possible to conduct banking transactions from a mobile device.

Customers of banks now have a wide variety of digital banking options, including online banking and mobile banking, that allow them to manage their money whenever and wherever they happen to be. E-banking, often known as Internet banking, provides users with access to their money and other financial services through an online website. Reference [4] “defined mobile banking as a channel whereby the customer interacts with a bank via a mobile device, such as a mobile phone or personal digital assistant.” When compared with internet banking, m-banking is not a supplement, but rather a digital alternative to traditional banking methods [5]. Increased smartphone use opens the door for more people to use mobile banking. The increasing availability of smartphones and the government’s promotion of the rise of electronic payments present an opportunity for India’s banking industry to rapidly expand its usage of mobile and internet banking. M-banking users worldwide reached 1.75 billion in 2019, and India has seen a substantial increase (Juniper Research 2019). The government of India mandated that all Indian banks offer M-banking to all of their customers by the end of 2021 in an effort to boost the country’s adoption of digital transactions. The “Reserve Bank of India (RBI)” is now implementing a bank-led strategy for mobile banking in India [6].The RBI has eliminated daily constraints on transactions for mobile banking users. There is a tremendous need for this kind of banking in India’s rural areas, where conventional financial institutions are few. Smartphones are widely accessible and inexpensive in India, allowing the vast majority of the country’s rural population to use them to connect to the web. As a result, mobile banking offers excellent prospects for India’s banks, telecom companies, and the Reserve Bank of India to reach rural India’s underbanked populace. When compared with China and the United States, India is anticipated to have the highest rate of growth in the value of digital transactions by 2023 [7].

Despite the fact that digital clients present a huge opportunity for banks to grow their customer bases, if they do not improve their customers’ experiences with digital banking channels, they risk losing those customers to rivals such as M-wallet service providers [8]. M-banking in India is still in its infancy, but online banking has already established itself as the dominant platform. It is clear that smartphone internet usage and penetration far exceed that of desktop and laptop PCs [9]. Mahmoud [10], Tran (2016), Oruç (2017), and Hamidi and Safareeyeh (2019) are just a few of the researches that have looked at the prevalence of digital banking in both established and developing nations. The m-banking industry has not really reached maturity; it has also not widely been adopted or spread in proportion to the completion of banking activities all over the world, particularly in developing countries [11]. Similarly, many researches have looked at why people who bank online are not using their phones. However, there is a dearth of research that directly addresses this issue in India.

This article aims to investigate the main drivers of mobile banking among Delhi–NCR consumers. The TAM (technological acceptance model) was used as a framework for this study. Only a few studies have looked at how online banking users in India plan to use other similar services, such as m-banking. In order to do this, a theoretical model was made using the technology acceptance model. This model was then expanded to include the factors that make m-banking users more likely to use mobile banking. These adoption factors include the feeling of being watched, the ability to do things on your own with a mobile device, social dominance, and the role of customer support as a mediator. The use of m-banking is the thing that matters.

In the last two decades, digital mobile devices have become the primary preferred method of consumer communication. Throughout the past year, mobile banking has become increasingly popular. The increasing number of smartphones in use, as well as the government’s push for cashless transactions, provide an opportunity for the Indian banking industry to rapidly expand its usage of mobile and online banking. There is a tremendous need for this kind of banking in India’s rural areas, where conventional financial institutions are few. Smartphones are widely accessible and inexpensive in India, allowing the vast majority of the country’s rural population to use them to connect to the web. As a result, mobile banking offers excellent prospects for India’s banks, telecom companies, and the Reserve Bank of India to reach rural India’s underbanked populace. When compared with China and the United States, India is anticipated to have the highest rate of growth in the value of digital transactions by 2023.

While mobile banking apps might be helpful, internet banking provides access to many more features, functions, and services that may be unavailable on mobile devices. With a simple text message, you can access your mobile bank. In order to use an online bank, you need a laptop or desktop computer and access to the internet. In recent years, the rate of global digitization has accelerated. In today’s fast-paced society, it is nearly impossible to survive without a smartphone. The introduction of mobile applications has altered the entire financial environment and made a variety of banking operations accessible from anywhere. Several years ago, users questioned the utility of mobile banking. Today, the situation has entirely turned, with more customers joining mobile banking apps than ever before. They switch to mobile banking for a seamless, hassle-free user experience and immediate account access.

## 2. Theoretical Background 

The theoretical framework for this investigation was drawn from the TAM [12]. This model’s primary advantage is that customers’ wants and needs can be determined to utilize all sorts of tech related to data storage and retrieval. It argues that an individual’s propensity to make use of technology depends on five factors unique to the information system (IS) environment: perceived utility, perception of usability, attitude, desire to use, and actual use [13]. The TAM model has been developed further by researchers who have added new dimensions, combined it with other theories, investigated various mediators, and identified the causes of perceived superiority in terms of utility and usability. When it comes to information systems, m-banking is brand new, cutting-edge, and sophisticated [14]. This research thus extends the TAM by incorporating the dimensions of sense of comfort of use, self-reliance, perceived surveillance, and customer assistance to comprehend customers’ behavioral intention to use m-banking. If an innovative kind of electronic banking, such as m-banking, is accessible and users feel confident in their ability to utilize it, existing banking clients are more willing to give it a try. M-banking apps need to be safe and immune to assaults such as phishing, spyware, and data leakage in order to attract and retain customers [15]. Customers who use mobile banking are likely to do their research before committing to a new banking channel, including reading reviews, researching the channel, consulting the channel’s FAQ, and consulting with their bank for help and special offers. Previous research has shown that variables including user friendliness, independence, surveillance, and social influence are crucial to the widespread usage of m-banking.

Technical advancements have transformed the scale and nature of the financial business, allowing it to grow beyond the conventional to the contemporary concept of saving and borrowing by way of technological development in the banking industry [16]. These terms allude to the inter-services between customers and bankers. The evolution of m-banking is the next step in the field of electronic banking; it allows clients to conduct banking transactions from the convenience of their mobile devices, rather than needing to physically visit their bank branch [17]. These days, bankers and their customers communicate via SMS or the mobile internet to improve customer satisfaction and loyalty by making banking available around the clock (24/7) and to save money on overhead while providing superior service to that offered by traditional branch locations (lower handling fees, fewer branches, fewer employees).

There are times where a mobile device is used to arrange, approve, and finalize the transfer of funds in exchange for products or services. Any device that can make a connection to a mobile telecommunication network and process a payment is considered a mobile device [18]. The bank delivers m-banking services to their customers with the desire to expand their customer share by overcoming all the impediments in the way of the adoption of m-banking services. The importance of banking to the operation of business and industry as a whole cannot be overstated. As Internet banking is still in its development stage, m-banking has evolved as the next advance way of doing banking [19]. A few examples of what can fall under the umbrella of “banking services” are the ability to make deposits, withdrawals, and other account adjustments, as well as having access to individualized data. Broadly speaking, “m-banking” refers to any situation where a consumer employs mobile communication techniques in combination with a mobile device to carry out financial services as part of an electronic operation [20].

Even banks and organizations are adopting “green” principles into their services and are committed to sustainability as a result of mounting pressure from shareholders, investors, and consumers. As a result, the banking sector is becoming a major influence and driving factor in sustainability. If your financial institutions have not already done so, reducing paper usage is one of the easiest ways to reduce their carbon impact. Consider that banks use a substantial amount of paper in their daily operations, including for service offers, customer service interactions, card statements, and more. Not only do banks have a quicker reaction time for mobile client contacts, but becoming paperless also improves efficiency and reduces operational expenses, such as document printing and storage. In addition, clients favor this strategy due to its easiness.

## 3. Review of the Literature

Although a modest beginning, mobile banking has flourished and transformed the way customers connect with businesses on a daily basis. According to [21] “there are not many interventions that have changed the business of banking as quickly as the e-banking revolution”. The academic community has taken notice of its recent growth and accomplishments, and, as a result, there are now a number of scholarly publications devoted to the topic [22]. To a large extent, the success of the Internet contributes to the widespread adoption of online and mobile banking. At the beginning, it was shown to be extremely useful for the banks on their own. This is due to the many advantages discussed herein, such as a smaller need for on-site staff, cheaper service fees, and more [23]. The approach has been so successful because it provides numerous advantages to consumers. As a result, it becomes clear that parties on either end of the transaction profit from using an online banking system [24].

Nearly everyone in the globe now has a mobile phone and relies on it often. Mobile banking is especially popular in India, where the number of people who use mobile phones greatly outnumbers those who use fixed lines [25]. This is why mobile phone infrastructure is more developed than landline phone infrastructure. In the financial business, technology plays a vital role [26]. The mobile phone is a ubiquitous technology item that has become a part of everyone’s life in the digital age [27]. Mobile banking is a burgeoning alternative delivery channel for banking services [28]. India is the second largest market for telecommunications in the world and has a great deal of potential for the expansion of mobile banking services [29].

Customer satisfaction has been shown to increase as a consequence of internet banking’s many benefits, including its simplicity of use, improved efficiency, and lower costs. Customers are more likely to stick with a bank if they have a positive experience using its services [30]. It was also discovered that new subsets of the population can be linked together. This is extremely helpful for consumers who live far from the nearest bank, since it will save them time and travel money [31]. Banks can save money by supplying less funding for traditional brick-and-mortar institutions because of the declining need for these organizations. There is no longer any need for a cashier to complete routine transactions considering they can all be completed digitally [32]. Along with the unexpectedly high rate of turnover, banks have also become more efficient [33]. Based on these merits, online banking has progressed steadily throughout the years.

Despite the concept’s obvious advantages and benefits, research shows that a sizable minority of bankers actually make use of the online channel. First, many customers still view traditional banking methods as equally vital, despite the rise of mobile banking [34]. Furthermore, some customers prefer the security of meeting with a professional in person, which is not accessible with mobile banking [35]. In addition, numerous writers raise concerns about the safety of using online and mobile banking, which may discourage some customers [36]. As with every new idea, there is a “switching cost” involved in initially adopting it [37]. However, banks might reap benefits from this switching cost if it leads to enhanced client loyalty [38]. Customers who feel more committed to a brand are more likely to make repeat purchases over the course of their lifetime, which should boost a company’s bottom line [39]. When it comes to the drawbacks of online banking, [40] cites a poor software design and implementation, faulty technologies and usability challenges, and consumer driven failure as the three main unsatisfactory features [41].

### Research Objectives

The objectives of the study are as follows:

1. To study if self-reliance has an effect or influence on customers’ mobile banking adoption.
2. To analyze if social domination has an effect on customers’ mobile banking adoption.
3. To study if perceived surveillance has an effect on customers’ mobile banking adoption.
4. To analyze if customer support has an influence on customers’ mobile banking adoption.

Research Gap: When it comes to information systems, m-banking is brand new, cutting-edge, and sophisticated [14]. This research thus extends the TAM by incorporating the dimensions of sense of comfort of use, self-reliance, perceived surveillance, and customer assistance to comprehend customers’ behavioral intention to use m-banking. If an innovative kind of electronic banking, such as m-banking, is accessible and users feel confident in their ability to utilize it, existing banking clients are more willing to give it a try. M-banking apps need to be safe and immune to assaults such as phishing, spyware, and data leakage in order to attract and retain customers [15]. Customers who use mobile banking are likely to do their research before committing to a new banking channel, including reading reviews, researching the channel, consulting the channel’s FAQ, and consulting with their bank for help and special offers. Previous research has shown that variables including user friendliness, independence, surveillance, and social influence are crucial to the widespread usage of m-banking.

## 4. Hypotheses Development

### 4.1. Self-Reliance

Mobile banking is an innovation digital banking channel that requires technical knowledge to use the mobile banking app. Mobile independence ideals are crucial for a new user to try out this channel. Self-reliance is defined as the “conviction that one can successfully execute the behavior required to produce the outcomes” [42]. Further, it states that “expectations of self-reliance determine whether coping behavior will be initiated, how much effort will be expended, and how long it will be sustained in the face of obstacles and aversive experiences” [43]. The impact of the conviction in one’s own ability to solve problems independently on future behavior has been the subject of previous studies. Whenever a customer does not think they have the technological know-how to use an m-banking service, they will not bother. This leads us to postulate the following. The following hypotheses are therefore proposed:

**H1:**  *Self-reliance has a significant influence on customers’ mobile banking adoption.*

**H2:**  *Customer support plays a mediating role between self-reliance and customers’ mobile banking adoption.*

### 4.2. Social Domination

Dominance in social situations is when one person exerts influence over another by influencing their behavior. Subjective norms, as presented by [44], are presented as forms of social dominance originating from established theories of IS. M-banking is a cutting-edge innovation that complements traditional online banking clients; thus, they are likely to look for reviews written by others who have used the service before making a decision to switch. Social dominance has been identified as a key predictor of intent to use in the existing research of mobile and online banking [45]. Hence, the following hypotheses are proposed:

**H3:**  *Social domination has a significant effect on customers’ mobile banking adoption.*

**H4:**  *Customer support plays a mediating role between social domination and customers’ mobile banking adoption.*

### 4.3. Perceived Surveillance

Existing research has shown that consumers’ behavioral intent to use internet banking is impacted by the degree to which banks monitor their use of the service [46]. Mobile banking’s slow penetration can be attributed in large part to customers’ worries about security [47]. A customer’s perception of risk when accessing m-banking increases if there are not sufficient surveillance mechanisms in place to protect them from the different surveillance concerns posed by a mobile device that provides banking services. This leads us to hypothesize the following:

**H5:**  *Perceived surveillance has a significant effect on customers’ mobile banking adoption.*

**H6:**  *Customer support plays a mediating role between perceived surveillance and customers’ mobile banking adoption.*

### 4.4. Customer Support 

Customer support consists of guidelines, frequently asked questions, help sites, and individual assistance [48]. Due to the widespread adoption of mobile banking, consumers now have a convenient digital banking option, including existing customers, and users anticipate support and rapid service prior to and after using the service [49]. As previous research has shown, customer service is a crucial factor in determining whether or not a given service will be adopted by its target audience. Hence, the following hypothesis is proposed:

**H7:**  *Customer support has a significant influence on customers’ mobile banking adoption.*

## 5. Research Methodology 

The present study is descriptive cum cross-sectional in nature while examining the role of self-reliance, social domination, perceived surveillance, and customer support with respect to the adoption of mobile banking. The primary data were gathered from the sample consumers residing in the “Delhi–NCR region”. In order to collect first-hand or original data from the customers, a modified web questionnaire divided into two parts was used by the researchers. Section-A of the questionnaire consists of various questions that describe the participants’ experiences of mobile banking services and Section-B comprises the questions that were asked about the consumers’ perceptions and observations towards mobile banking in today’s era of digitalization. The statements used in the research study to collect the sample dataset were further grouped into five major latent constructs, i.e., self-reliance, social domination, perceived surveillance, and customer support with respect to the adoption of mobile banking. All of these statements [based on “five-point Likert Scale” ranging from “strongly disagree (1) to strongly agree (5)”] were designed by the researchers, keeping in mind that they were all asked in the context of respondents’ relationship with mobile banking services. 

The study was conducted from July to October 2022 on 376 sample consumers who widely used mobile banking services in Delhi–NCR. For this study, the researchers had more information about the knowledge and understanding of the people they selected to participate in the study. That assured that the researchers would have the desired number of sample customers to choose from when conducting their research. Therefore, the “judgmental” (or purposive) sampling technique was conducted by the researchers to obtain the required responses from the consumers. For the analysis of the collected sample dataset, descriptive statistics were conducted through SPSS software in the study to classify the demographic profiles of the sample consumers. Structural equation modeling (SEM) was applied to examine whether there was a significant relationship between all the study variables with the help of SmartPLS 3.0 software. Furthermore, the “Mann-Whitney U Test” and the “Kruskal–Wallis H Test” were also used for analyzing the demographic variables of the study.

## 6. Background Information of the Respondents

This portion displays the sample participants of the individuals who completed the questionnaire. Table 1 displays the responses to the questionnaires on the selected demographic variables for the study. The information presented here was obtained from primary data.

Table 1 denotes the participants’ demographic statistics based on their gender, age group, educational qualification, occupational status, and monthly income. It shows that 54.25% of the sample respondents were males (M), whereas 45.74% were females (F). The following detail indicates that 25% of the respondents fit the age group of 31–40 years, 41.22% were between 21 and 30 years, 16.75% belonged to the age group above 40 years, and, lastly, 17.02% fell within the age group of up to 20 years.

Educational qualification indicates that 17.8% of the respondents represented undergraduates (U.G), 28.45% were graduates (G), 15.69% were post graduates (P.G), 9.57% represented Ph.D. and 28.45% represented professional degree holders (PDH). Occupational status illustrated that 13.56% of respondents represented government employees, 43.35% were private employees, 28.72% were business or self-employed, and 14.36% represented students.

The monthly income of the respondents shows that 19.14% respondents represented an income of INR ≤ 10,000, 36.43% respondents had an income of INR 10,000–20,000, 24.73% of respondents had an income of INR 20,001–40,000, and 19.68% of respondents had an income of INR < 40,000.

### 6.1. Measurement Model Evaluation

The measuring model was tested using internal consistency, convergent validity, and discriminant validity. 

Table 2 shows that the mean values of all the items in each construct are greater than 3. It indicates the perceived surveillance, self-reliance, social domination, and customer support. In this study, the researchers used the “five-point Likert scale,” ranging from “strongly disagree (1) to strongly agree (5)”. The factor loadings of all the items in each construct are greater than the prescribed limit of 0.70. Therefore, it indicates that all the statements clearly explain their respective theoretical assumed construct.

**Figure 1:**
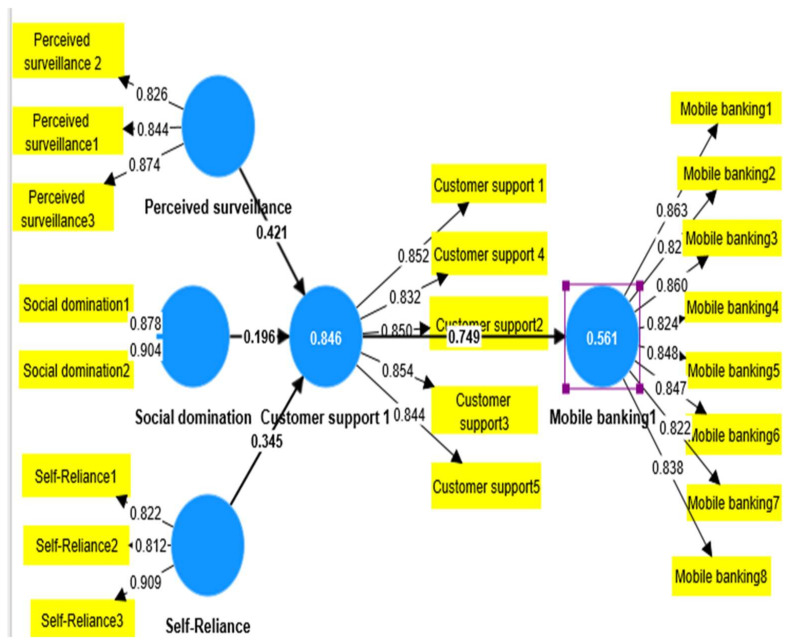
Shows factor loading, beta values, and R-square of items and construct.

In Figure 1, perceived surveillance, self-reliance, social domination, and customer support and mobile banking are represented by circles since they are the latent constructs used by the researchers in the study [50]. Perceived surveillance was measured through three statements codes as perceived surveillance one to three, self-reliance was measured through three statements codes as self-reliance one to three, social domination was measured through two statements codes as social domination one to two, customer support was measured through five statements codes as customer support one to five, and mobile banking was represented through codes as mobile banking one to eight. The factor loading values are shown near the arrows pointing to the respective items/constructs [51]. The factor loading values are shown near the arrows pointing to the respective items/constructs. Regression value, also in Figure 1, shows the regression weight in the circle of the construct.

Table 3 clearly shows that all four constructs met the required thresholds limit, as the value of composite reliability (C.R) was above 0.7 and average variance extracted (AVE) exceeded 0.5 [52]. The value of Cronbach’s alpha and rho-a value established internal consistency and were also greater than 0.7 [53]. Therefore, the convergent validity of the constructs was proved [54].

### 6.2. Discriminant Validity Result

The Fornell–Larcker and cross-loading criteria were examined to check the discriminant validity. Discriminant validity indicates “the extent to which the measure is adequately distinguishable from related constructs within the nomological net”.

Table 4 represents the Fornell–Larcker criterion. In this criterion you take the square roots of average variance extracted of the available constructs. The values were as follows: perceived surveillance (0.791), self-reliance (0.818), social domination (0.841), customer support (0.831), and mobile banking (0.891), which exceeded the correlation values found between any two constructs and any three or more constructions. According to the Fornell–Larcker test, discriminant validity was thus demonstrated [55].

Table 5 displays the cross-loading criterion where all construct loadings are greater than cross-loadings with other constructs across columns. According to the cross-loading criterion, we have established that our test is legitimate in its ability to discriminate [56].

### 6.3. Structural Model Evaluation

Multicollinearity should be checked whenever the structural model is being evaluated to assure reliable outcomes. Results from 1.519 to 2.138 for the variance inflation factor (VIF) suggest that the model had no multicollinearity [57]. The validity of the hypotheses were then evaluated by subjecting the structural model to a bootstrapping test (3000 resamples).

**Figure 2:**
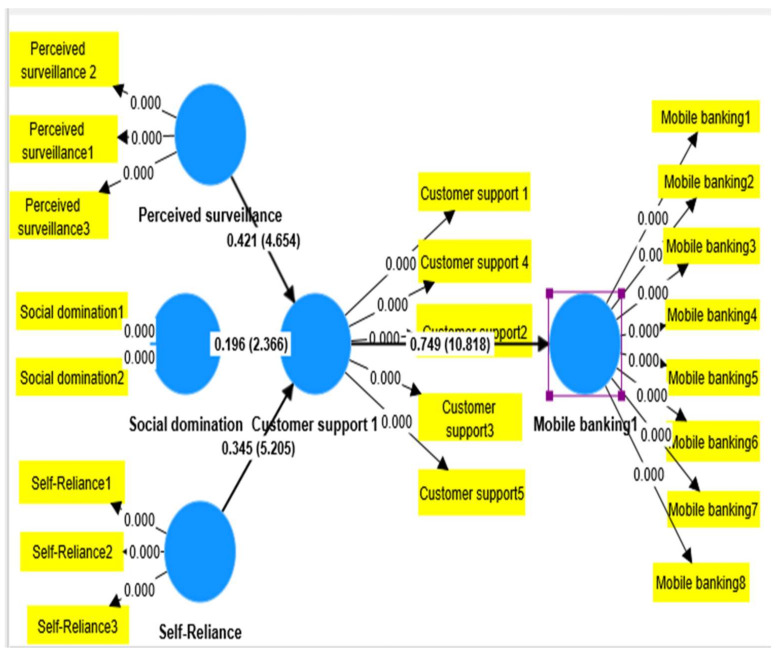
Shows t-values of all items and constructs.

When the t-values of the regression weights are more than the maximum of 1.96, as shown in Figure 2 of the preceding PLS-SEM model, it can be concluded that the path in question is statistically significant at the 5% level [58]. Table 6 displays the SEM model’s derived results.

Table 6 shows that hypotheses Ho1 and Ho5 were supported, and that self-reliance directly and positively related to mobile banking adoption (β = 0.597, t-value = 4.361, and *p* < 0.001). Perceived surveillance directly and positively related to mobile banking adoption (β = 0.351, t-value = 2.641, and *p* < 0.001). 

Table 6 also shows that hypotheses H3 and H7 were not supported, and that social domination was not directly and positively related to mobile banking adoption (β = −0.008, t-value = 0.068, and *p* ≥ 0.001). Customer support was not directly and positively related to mobile banking adoption (β = 0.066, t-value = 0.311 and *p* ≥ 0.001).

Table 7 shows that hypotheses Ho2, H04, and Ho6 were supported, and that customer support showed mediation or indirect effect on self-reliance and mobile banking adoption (β = 0.282, t-value = 3.613, and *p* ≤ 0.001). Customer support showed mediation or indirect effect on social domination and mobile banking adoption (β = 0.229, t-value = 2.134, and *p* ≤ 0.001). Customer support showed mediation or indirect effect on perceived surveillance and mobile banking adoption (β = 0.465, t-value = 4.257, and *p* ≤ 0.001). In Figure 2, all hypothesis values are mentioned.

Table 8 was used to determine the statistical disparity between demographic characteristics using the “Mann-Whitney U Test” and the “Kruskal–Wallis H Test. In the case of gender, there were no significance differences of self-reliance, social domination, and perceived surveillance except customer support (*p* ≤ 0.001). In the case of age, there were no significance differences of customer support, social domination, and perceived surveillance except self-reliance (*p* ≤ 0.001). In the case of educational qualification, there were no significance differences of social domination, perceived surveillance, and customer support.

In the case of income, there were no significance differences of social domination, perceived surveillance, and customer support except perceived surveillance (*p* ≤ 0.001). In the case of occupational status, there were no significance differences of social domination and perceived surveillance except customer support (*p* ≤ 0.001).

## 7. Implications

The consequences of this study’s findings are diverse. Social dominance and perceived surveillance have a beneficial influence on the technology adoption of online banking, whereas self-reliance has no positive effect on the technology adoption of mobile banking. This study adds to the literature by including the relative advantage, perceived surveillance, and sense of ease of usage as observed variables for potential advantages. In the same way, concerns of trust, surveillance, and the law were taken into account when discussing self-reliance, while issues of subjective norm and image were considered while discussing social dominance. Managers must therefore dispel the myth that online banking is subject to enhanced supervision. The research shows that the main issues that managers should be concerned with while adopting mobile banking are those that customers believe to be under monitoring, such as the safety of their data and transactions and the legal protections afforded to them. Customers that care about their reputations and keeping up with the latest fashions are the ones who are most likely to be interested in switching to online banking, as shown by this research. This suggests that a region with a sizable young population is more likely to embrace mobile banking.

The majority of customers perceived ‘privacy and security’ as a critical issue. Here, banks are advised to educate customers on this issue to raise their awareness. Especially for the customers’ worries such as losing money if one’s mobile handset is lost (a substantial number of respondents worried about this). Secondly, banks and telecom operators are suggested to draft comprehensive joint policies regarding security and privacy so that customers can be assured at both the bank and telecom operator levels while doing mobile banking.

‘Standardization’ is another major issue, as a lack of standardization of mobile banking services in the country resulted in increased complexity while using mobile banking services (especially when using the mobile banking services of multiple banks). Even banks and organizations are adopting “green” principles into their services and are committed to sustainability.

## 8. Conclusions

The primary purpose of this research is to learn what aspects of mobile banking are driving its popularity. Even though it is becoming more common, m-banking is still in its infancy in India; therefore, research into what variables encourage or discourage consumers from using the service is essential. As a result of this study, online bankers have a better idea of where they should focus their efforts in order to create reliable mobile banking services, and they have guidelines for making better banking decisions in the future by carefully considering possible obstacles. This study filled a gap in the literature by determining what characteristics affect consumers’ willingness to use m-banking services. This study built a theoretical framework to identify and analyze the elements that influence consumers’ decisions to switch to mobile banking among those who already use mobile banking. The highlighted parameters provide a holistic perspective on the primary causes driving plans for using mobile banking and the features that need to be incorporated to boost adoption. This work fills a large gap in the literature by elaborating on these connections.

## 9. Limitation and Future Research

The study’s results are restricted by a variety of caveats. Firstly, the sample techniques used in the study introduce a limitation to the results’ generalizability. Samples were acquired from both customers and employees of banks. Therefore, even though non-random sampling was utilized, it was not completely random. Those who received the opinion questionnaire were typically frequent Internet users, and hence were likely to also be active mobile banking users. Furthermore, data collection was performed manually in one place in India. Furthermore, they may not reflect the entire Indian population, and future studies may want to explore using a greater sample size. Additionally, there may be a bias issue with the selection of samples when using this method of convenience sampling. Research with a larger sample of customers is required to determine whether or not this model is applicable, as the current sample is too small to draw any firm conclusions. Future research employing a longitudinal study design could examine this research model at several time points and make comparisons, focusing attention on the mobile banking phenomenon.

## Figures and Tables

**Table 1:** Baseline Data of the Participants (N= 376).

Basis	Categories	F	C.F	%
**Gender**	M	204	204	54.25
	F	172	376	45.74
**Age** **Group**	Up to 20 years	64	64	17.02
	21–30 years	155	219	41.22
	31–40 years	94	313	25
	41 and above	63	376	16.75
**Educational Qualification**	U. G	67	67	17.8
	G	107	174	28.45
	P. G	59	233	15.69
	Dr	36	269	9.57
	P.D.H	107	376	28.45
**Occupational** **Status**	Govt. Employees	51	51	13.56
	Private Employees	163	214	43.35
	Business and Self Employed	108	322	28.72
	Students	54	376	14.36
**Monthly** **Income**	≤INR 10,000	72	72	19.14
	INR 10,000–INR 25,000	137	209	36.43
	INR 25,001–INR 50,000	93	302	24.73
	>INR 50,000	74	376	19.68

**Table 2:** Mean, SD, and loadings of constructs.

Construct	Item	Mean	SD	Loading
**Perceived surveillance**	Perceived surveillance1	4.14	0.846	0.801
	Perceived surveillance2	4.13	0.866	0.757
	Perceived surveillance3	4.26	0.885	0.811
**Self-reliance**	Self-reliance1	4	0.795	0.764
	Self-reliance2	4.2	0.841	0.826
	Self-reliance3	4.095	0.847	0.863
**Social domination**	Social domination1	3.89	0.71	0.76
	Social domination2	3.54	0.68	0.74
**Customer support**	Customer support1	4.089	0.854	0.825
	Customer support2	4.137	0.841	0.822
	Customer support3	4.179	0.808	0.838
	Customer support4	4.253	0.858	0.839
	Customer support5	3.90	0.71	0.81
**Mobile banking**	Mobile banking1	4.126	0.798	0.797
	Mobile banking2	4.076	0.761	0.874
	Mobile banking3	4.189	0.858	0.819
	Mobile banking4	3.14	0.746	0.821
	Mobile banking5	4.43	0.766	0.777
	Mobile banking6	4.16	0.815	0.821
	Mobile banking7	4.01	0.715	0.864
	Mobile banking8	3.21	0.821	0.846

**Table 3:** Convergent validity result.

Factor	Cronbach’s Alpha	Rho-A	C.R	A. V. E
Perceived surveillance	0.850	0.852	0.893	0.625
Self-reliance	0.834	0.839	0.890	0.669
Social domination	0.792	0.796	0.878	0.707
Customer support	0.856	0.821	0.899	0.691
Mobile banking	0.861	0.850	0.801	0.611

**Table 4:** Discriminant validity–Fornell–Larcker criterion.

Factors	Perceived Surveillance	Self-Reliance	Social Domination	Customer Support	Mobile Banking
Perceived surveillance	**0.791**	0	0	0	0
Self-reliance	0.754	**0.818**	0	0	0
Social domination	0.759	0.789	**0.841**	0	0
Customer support	0.773	0.734	0.749	**0.831**	0
Mobile banking	0.711	0.720	0.703	0.619	**0.819**

According to Fornell–Larcker criterion where all construct loadings are greater than cross-loadings with other constructs across columns. It is requiring to bold to create difference among other variables.

**Table 5:** Discriminant validity–loading and cross-loading criterion.

Factor	Perceived Surveillance	Self-Reliance	Social Domination	Customer Support	Mobile Banking
Perceived surveillance1	**0.801**	0.593	0.647	0.610	0.593
Perceived surveillance2	**0.757**	0.561	0.549	0.592	0.561
Perceived surveillance3	**0.811**	0.649	0.631	0.665	0.649
Self-reliance1	0.589	**0.764**	0.505	0.693	0.579
Self-reliance2	0.582	**0.801**	0.667	0.488	0.661
Self-reliance3	0.596	**0.720**	0.568	0.569	0.633
Social domination1	0.627	0.626	**0.826**	0.675	0.682
Social domination2	0.632	0.510	**0.863**	0.639	0.600
Customer support1	0.610	0.620	0.509	**0.814**	0.509
Customer support2	0.716	0.681	0.641	**0.825**	0.688
Customer support3	0.598	0.602	0.592	**0.822**	0.590
Customer support4	0.579	0.611	0.635	**0.838**	0.568
Customer support5	0.661	0.535	0.589	**0.839**	0.630
Mobile banking1	0.633	0.603	0.638	0.598	**0.797**
Mobile banking2	0.682	0.701	0.712	0.579	**0.874**
Mobile banking3	0.600	0.681	0.535	0.661	**0.849**
Mobile banking4	0.693	0.542	0.596	0.619	**0.797**
Mobile banking5	0.488	0.610	0.627	0.709	**0.874**
Mobile banking6	0.569	0.678	0.632	0.654	**0.849**
Mobile banking7	0.675	0.605	0.610	0.530	**0.791**
Mobile banking8	0.639	0.523	0.716	0.590	**0.810**

According to Fornell–Larcker criterion where all construct loadings are greater than cross-loadings with other constructs across columns. It is requiring to bold to create difference among other variables.

**Table 6:** Direct impact of service quality and hypothesis testing.

Hypothesis	Path	Β	t-Value	*p*-Value	Result
H1	Self-reliance → mobile banking adoption	0.597	4.361	*p* ≤ 0.001	Supported
H3	Social domination → mobile banking adoption	−0.008	0.068	*p* ≥ 0.001	Not Supported
H5	Perceived surveillance → mobile banking adoption	0.351	2.641	*p* ≤ 0.001	Supported
H7	Customer support → mobile banking adoption	0.066	0.311	*p* ≥ 0.001	Not Supported

**Table 7:** Mediation effect or indirect effect of satisfaction and hypothesis testing.

Hypothesis	Path	Β	t-Value	*p*-Value	Result
H2	Self-reliance → Customer support → mobile banking adoption	0.282	3.613	*p* ≤ 0.001	Supported
H4	Social domination → Customer support → mobile banking adoption	0.229	2.134	*p* ≤ 0.001	Supported
H6	Perceived surveillance → Customer support → mobile banking adoption	0.465	4.257	*p* ≤ 0.001	Supported

**Table 8:** Mann-Whitney U Test and Kruskal–Wallis H test—Demographic profile (see Appendix A).

Demographic Profile		Mean				*p*-Value			
		Self-Reliance	Social Domination	Perceived Surveillance	Customer Support	Self-Reliance	Social Domination	Perceived Surveillance	Customer Support
Gender	Male	88.77	95.08	85.87	89.31	0.811	0.32	0.507	*p* ≤ 0.001
	Female	86.90	77.88	91.04	86.13				
Age Group	Up to 20 years	90.21	94.64	107.29	83.86	*p* ≤ 0.001	0.236	0.467	0.18
	21–30 years	82.55	77.36	84.73	92.33				
	31–40 years	87.48	95.40	88.84	86.41				
	41 and above	97.30	86.55	83.42	86.06				
Educational Qualification	Undergraduate	93.09	87.82	102.64	102.64	0.158	0.727	0.678	0.22
	Graduate	71.57	94.70	92.43	85.19				
	Post Graduate	98.58	80.88	88.69	88.02				
	Doctor	87.84	92.52	77.96	83.08				
	Professional Degree	86.42	89.42	85.33	89.51				
Monthly Income	≤INR 10,000	90.57	78.38	90.76	83.00	0.297	0.595	*p* ≤ 0.001	0.20
	INR 10,000-INR 25,000	80.98	91.37	94.78	84.73				
	INR 25,001–INR 50,000	84.21	92.06	85.01	93.82				
	>INR 50,000	100.37	82.44	85.05	83.61				
Occupational Status	Govt. Employees	89.97	79.03	85.00	82.47	0.770	0.343	0.21	*p* ≤ 0.001
	Private Employees	83.70	83.26	86.72	93.81				
	Business and Self Employed	94.60	98.49	87.40	89.76				
	Students	81.86	83.75	97.75	86.18				

## Data Availability

The data used to support the findings of this study are available from the corresponding author upon request.

## Appendix A

**Part 1. Demographic Profile**

Name of Respondent:         E-mail-id:

1. Gender
  □ Male
  □ Female
2. Age Group
  □ Up to 20 years
  □ 21–30
  □ 31–40
  □ 40 and above
3. Educational Qualification
  □ Undergraduate
  □ Graduate
  □ Post Graduate
  □ Doctorate
  □ Professional Degree
4. Occupational Status
  □ Govt. Employee
  □ Private Employee
  □ Business/Self employed
  □ Student
5. Monthly income
  □ ≤INR 10,000
  □ INR 10,000–INR 25,000
  □ INR 25,000–INR 50,000
  □ >INR 50,000
6. You have an account in Delhi□
  □ Yes
  □ No
7. You using mobile banking services□
  □ Yes
  □ No

If yes, then proceed to next section.

**Part 2**

(Please put (✓) mark to your response on the scale of 1–5 depending upon your level of agreement or disagreement). **Where: (1 = Strongly Disagree, 2 = Disagree, 3 = Neutral, 4 = Agree, 5 = Strongly Agree).**

**S.** **No.**	**Statements**	**Strongly Disagree** **(1)**	**Disagree** **(2)**	**Neutral** **(3)**	**Agree** **(4)**	**Strongly Agree** **(5)**
**Factor 1. Perceived surveillance,**						
9.1	E-banking accessible 24 × 7					
9.2	E-banking provides a level of security and privacy that is unmatched.					
9.3	I am confident that the electronic banking system will act in my best interests.					
**Factor-2 Self-reliance**						
9.4	The e-banking website’s security measures safeguard the data I submit.					
9.5	I have no difficulty learning how to operate the e-banking system.					
**Factor-3 Social domination**						
9.7	I intend to utilize electronic banking on a regular basis in the future.					
9.8	I am pleased with the current e-banking maintenance fees.					
9.9	My overall experience with this website has been positive.					

(Please put **(✓)** mark to your response on the scale of 1–5 depending upon your level of agreement or disagreement). **Where: (1 = Strongly Disagree, 2 = Disagree, 3 = Neutral, 4 = Agree, 5 = Strongly Agree).**

**S.** **No.**	**Statements**	**Strongly Disagree** **(1)**	**Disagree** **(2)**	**Neutral** **(3)**	**Agree** **(4)**	**Strongly Agree** **(5)**
**Factor 4. Customer support**						
9.1	The bank’s reputation and size demonstrate its commitment to e-banking integrity.					
9.2	The e-banking website is simple to explore.					
9.3	The e-banking website features a simple inquiry process.					
9.4	I have no difficulty comprehending the information contained in this e-banking system.					
9.5	I have access to e-banking at any time and from any location in the world.					
**Factor 5. Mobile banking**						
9.6	I am pleased with the e-banking website’s transaction processing.					
9.7	I am comfortable with the e-banking website’s security features.					
9.8	I am satisfied with the e-banking service’s quality.					
9.9	I find the e-banking system to be quite adaptable in terms of interaction.					
9.10	The e-banking website features a simple inquiry process.					
9,11	I am pleased with the current e-banking maintenance fees.					
